# Radiosensitization to γ-Ray by Functional Inhibition of APOBEC3G

**DOI:** 10.3390/ijms23095069

**Published:** 2022-05-03

**Authors:** Ying Tong, Sota Kikuhara, Takae Onodera, Lichao Chen, Aung Bhone Myat, Shoji Imamichi, Yuka Sasaki, Yasufumi Murakami, Tadashige Nozaki, Hiroaki Fujimori, Mitsuko Masutani

**Affiliations:** 1 Department of Molecular and Genomic Biomedicine, Center for Bioinformatics and Molecular Medicine, Nagasaki University Graduate School of Biomedical Sciences, Nagasaki 852-8523, Japan; bb55320022@ms.nagasaki-u.ac.jp (Y.T.); takae-o@nagasaki-u.ac.jp (T.O.); chen202107@outlook.com (L.C.); aumiat.abm@nagasaki-u.ac.jp (A.B.M.); simamich@ncc.go.jp (S.I.); jj20210059@nagasaki-u.ac.jp (Y.S.); nozaki@cc.osaka-dent.ac.jp (T.N.); cell4thedition@yahoo.co.jp (H.F.); 2 Lab of Collaborative Research, Division of Cellular Signaling and Central Radioisotope Division, National Cancer Center Research Institute, Tokyo 104-0045, Japan; 8314623@ed.tus.ac.jp; 3 Department of Biological Science and Technology, Faculty of Industrial Science and Technology, Tokyo University of Science, Tokyo 162-8601, Japan; yasufumi@rs.noda.tus.ac.jp; 4 Department of Pharmacology, Faculty of Dentistry, Osaka Dental University, 8-1 Hirakata, Osaka 573-1144, Japan

**Keywords:** APOBEC3G, γ-irradiation, radiosensitization

## Abstract

The radiosensitization of tumor cells is one of the promising approaches for enhancing radiation damage to cancer cells and limiting radiation effects on normal tissue. In this study, we performed a comprehensive screening of radiosensitization targets in human lung cancer cell line A549 using an shRNA library and identified *apolipoprotein B mRNA editing enzyme catalytic subunit 3G* (*APOBEC3G: A3G*) as a candidate target. *APOBEC3G* is an innate restriction factor that inhibits HIV-1 infection as a cytidine deaminase. *APOBEC3G* knockdown with siRNA showed an increased radiosensitivity in several cancer cell lines, including pancreatic cancer MIAPaCa2 cells and lung cancer A549 cells. Cell cycle analysis revealed that *APOBEC3G* knockdown increased S-phase arrest in MIAPaCa2 and G2/M arrest in A549 cells after γ-irradiation. DNA double-strand break marker γH2AX level was increased in *APOBEC3G*-knocked-down MIAPaCa2 cells after γ-irradiation. Using a xenograft model of A549 in mice, enhanced radiosensitivity by a combination of X-ray irradiation and *APOBEC3G* knockdown was observed. These results suggest that the functional inhibition of *APOBEC3G* sensitizes cancer cells to radiation by attenuating the activation of the DNA repair pathway, suggesting that *APOBEC3G* could be useful as a target for the radiosensitization of cancer therapy.

## 1. Introduction

The importance of radiotherapy in cancer treatment [1] is expected to increase because of its high therapeutic efficacy and low invasiveness; however, accompanying damage to adjacent organs is an unavoidable risk. To enhance therapeutic effects while reducing adverse effects on neighboring organs, various methods have been continually improved and developed from physical and biological perspectives. The development of radiation sources and devices has progressed, as evidenced by advanced techniques such as intensity-modulated radiation therapy (IMRT) [2], utilization of charged particles, which release their energy with the production of a Bragg peak [3], use of high linear energy transfer (LET) beams such as heavy particle beams [4], and so on. From the biological viewpoint, the radioprotection of normal tissues and radiosensitization of tumor cells have been developed as promising approaches for increasing the efficiency of radiotherapy [5]. Anti-cancer agents targeting DNA synthesis, such as cisplatin [6] and 5-fluorouracil [7], have been also used as radiosensitizers for cancers in clinical practice. Their use in combination with radiation is highly effective but sometimes causes serious adverse effects. Low toxicity to normal cells is a required feature for radiosensitizers. To date, various types of radiosensitizing targets have been identified, for example, poly (ADP-ribose) polymerase (PARP) [8,9], poly (ADP-ribose) glycohydrolase (PARG) [10], checkpoint kinase 1 (CHK1) [11], heat-shock protein 90 (HSP90) [12], ataxia telangiectasia mutated (ATM) kinase [13], histone deacetylase (HDAC) [14], vascular endothelial growth factor (VEGF) [15], and DNA methyltransferase [16]. Some of their inhibitors have been evaluated in clinical tests [17,18].

Cancer tissues are heterogeneous and possess a wide variety of genetic alterations [19], which make it difficult to develop a radiosensitizer universally applicable to different types of cancer cells. Having a variety of therapeutic targets suitable to individual cancers is a critically important strategy in cancer therapy. Because radiotherapy is one of the basic methods of treatment in lung cancer, for this research we performed a comprehensive analysis of radiosensitization target genes in lung cancer cell line A549. Here in this study, our genome-wide screening identified *apolipoprotein B mRNA editing enzyme catalytic subunit 3G* (*APOBEC3G*: *A3G*) as a candidate. A3G, an innate restriction factor that inhibits human immunodeficiency virus-1 (HIV-1) infection, is a cytidine deaminase that catalyzes the deamination of cytosine to uracil in the single-stranded DNA substrate and restricts retroviral replication by inducing hypermutation in viral nascent reverse transcripts [20]. Recent studies suggested that not only does it contribute to the prevention of viral infection, A3G also has important non-antiviral functions. The upregulated *A3G* expression is related to a poor prognosis in colorectal cancer with hepatic metastasis by promoting cancer cell migration and invasion through inhibiting the *miR-29*-mediated suppression of metastasis activator matrix metalloprotease 2 (MMP2) [21,22]. Moreover, A3G enhances tumor resistance to radiotherapy in lymphoma and glioblastoma cells via the activation of the DNA repair pathway [23,24]. These results suggest the potential of A3G as a target for sensitization to radiotherapy in cells expressing high A3G levels. In this study, we showed that *A3G* knockdown induces radiosensitization in various kinds of cell lines, and in a xenograft model. Our study supports the notion that *A3G* is a potential target for inducing radiosensitization.

## 2. Results

### 2.1. Comprehensive Analysis to Identify Radiosensitization Targets

**Figure 1:**
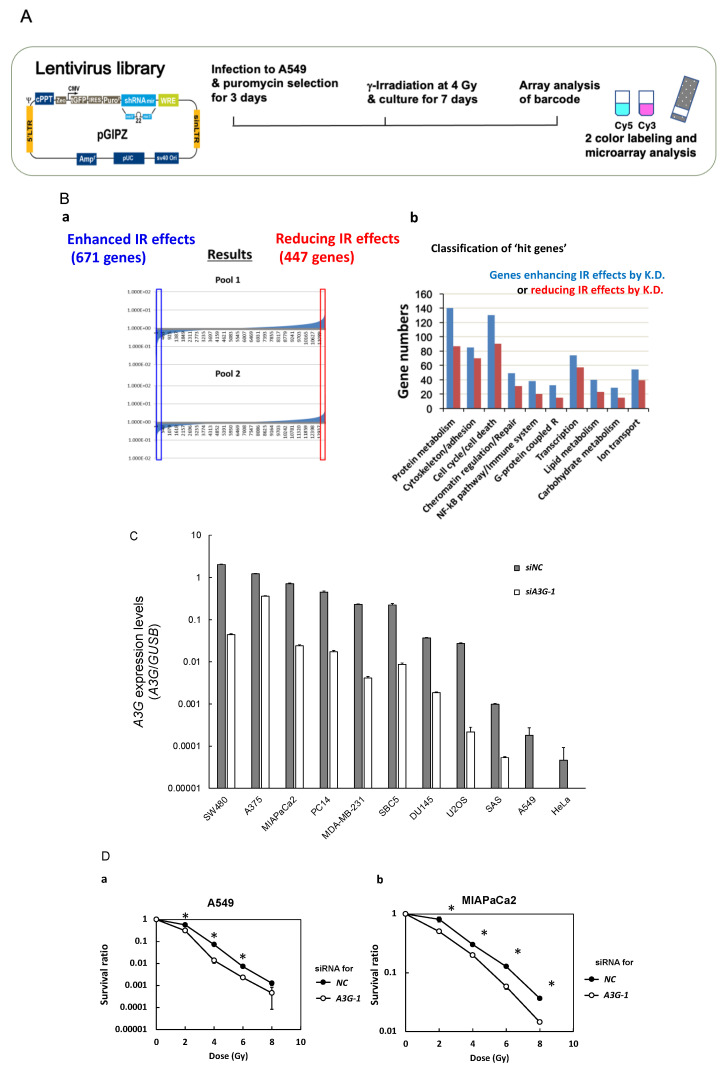

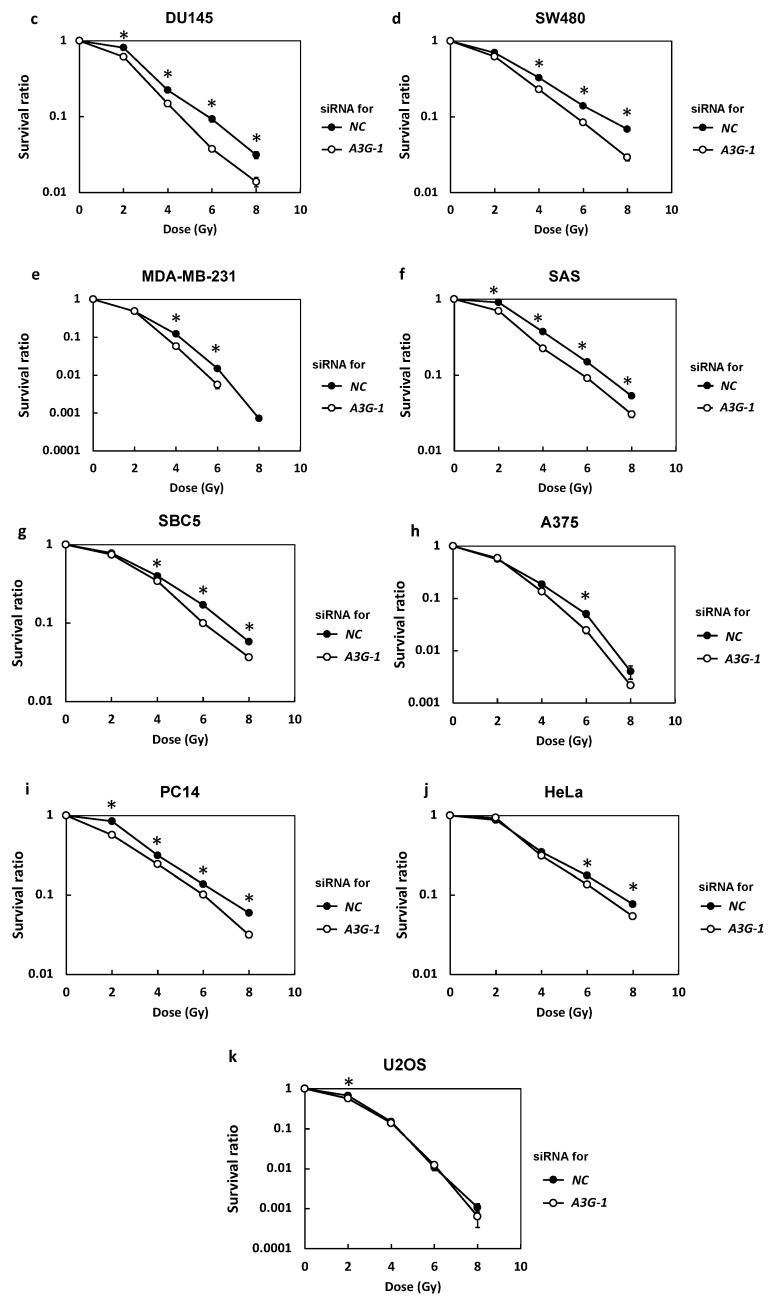
Negative screening of radiosensitization targets using an shRNA library. (**A**) Lung cancer A549 cells were infected with a lentiviral shRNA expression library and γ-irradiated at 4 Gy or mock-irradiated, and then cultured for seven days. Candidate genes involved in radiosensitization and radioresistance were identified by microarray analysis using barcode sequences as described in Materials and Methods. (**B**) a. The results of negative screening using pool1 and pool2. b. Classification of hit genes is shown separately for enhancing ionizing radiation (IR) effects and reducing IR effects. (**C**) Relative *A3G* mRNA expression levels and knockdown levels analyzed by real-time PCR analysis in 11 cancer cell lines. *A3G* expression in cancer cell lines were compared using a primer set that detects all main reported splicing variants (see Figure S1). *A3G* gene was knocked down with si*A3G-1*, which targets the common exon sequence present in all main splicing variants. (**D**) a–k. Comparison of the effect of *A3G* knockdown on radiosensitization in 11 cancer cell lines from various cancer types analyzed after γ-irradiation by colony formation assay.

To identify new target genes for which the loss of expression enhances radiosensitivity, genome-wide negative screening with a lentiviral shRNA library containing approximately 10,000 genes was performed. For this purpose, we used lung cancer cells, because radiation therapy is frequently used for various types of lung cancers from early to progressed stages. Human lung adenocarcinoma A549 cells were transfected with a lentiviral shRNA library, then divided into two groups: one of which was γ-irradiated at 4 Gy, the other was mock-irradiated, and both were then cultured for 7 days. To quantify differences in the abundances of each shRNA clone between non-irradiated and irradiated cells, genomic DNA were extracted from each cell group and specific barcode sequences incorporated into genomic DNA were amplified by PCR and analyzed using microarray. The declined detection in γ-irradiated cells containing a specific shRNA indicates that the knockdown of the corresponding target gene caused radiosensitivity (Figure 1A).

Using a 2-fold difference between irradiated and non-irradiated cells as a threshold, we identified 671 sensitizing and 447 resistance-inducing candidate genes (Figure 1(Ba)). These candidate genes contained protein metabolism, cell cycle, and cell-death-related genes, which are consistent with the literature describing how genes related to DNA repair, cell cycle checkpoint, and cell death are a potential target for radiosensitization (Figure 1(Bb)).

To validate these radiosensitizing candidate genes, we performed a secondary screen using 29 picked up genes which were expected to show radiosensitizing effects. Among these genes, we focused on one candidate gene, *A3G*, because A3G is involved in DNA repair, and so targeting *A3G* can be expected to sensitize cancer cells to ionizing radiation by attenuating the activation of the DNA repair pathway.

### 2.2. Radiosensitization Profiles in Cancer Cell Lines by A3G Knockdown

To assess whether the knockdown of the *A3G* gene induces radiosensitization, the survival rates of *A3G*-knockdown and control cells exposed to γ-irradiation were compared in 11 solid cancer-derived cell lines. As shown in Figure 1C, *A3G* expression levels were diverse between cell lines. Whereas *A3G* levels were reported to correlate with radiosensitivity in lymphoma cells [23], there was no correlation between *A3G* expression levels and radiosensitivity among cells tested in our experiment (data not shown).

*A3G* downregulation caused increased radiosensitivity in 10 cell lines tested, except for U2OS cells (Figure 1(Da–Dk)). Radiation dose enhancement ratios for survival at 10% (ER_10_) are summarized in Table 1. ER_10_ ranged from 1.35 to 1.01, with A549 cells showing the highest value, although its basal expression level was low. Among 11 cell lines tested, there was no correlation between *A3G* expression level and radiosensitizing efficiency.

### 2.3. Enhanced S-Phase Arrest and Defect in DNA Damage Response by A3G Knockdown in MIAPaCa2 Cells

**Figure 2:**
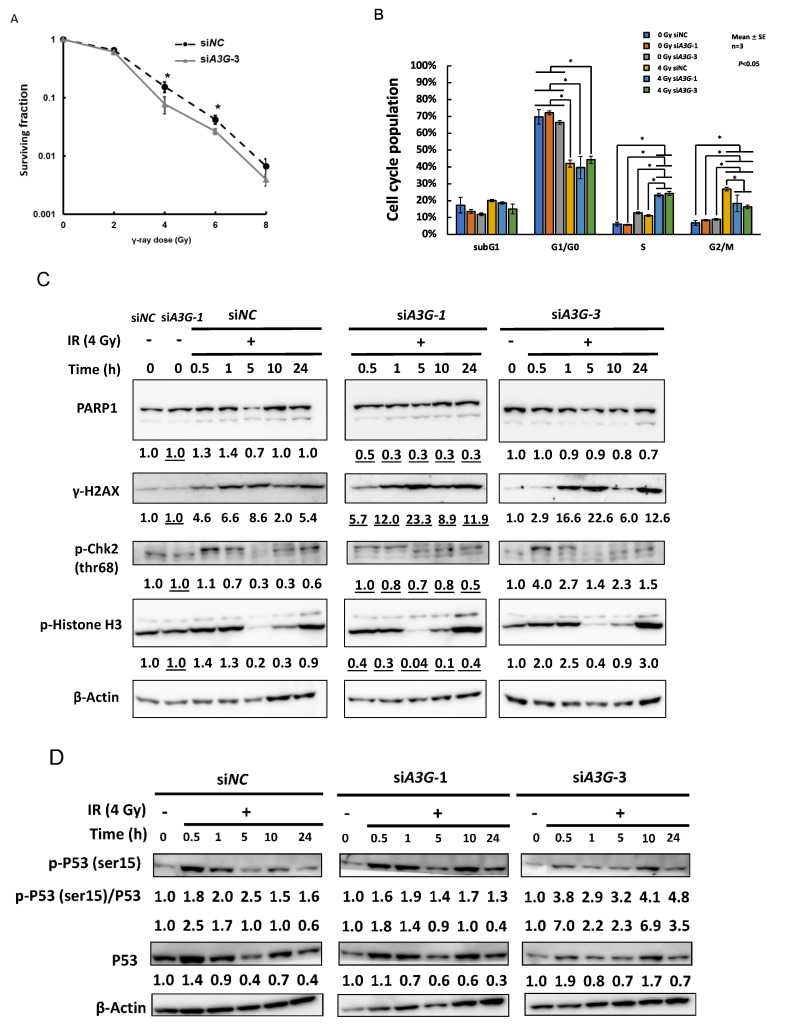

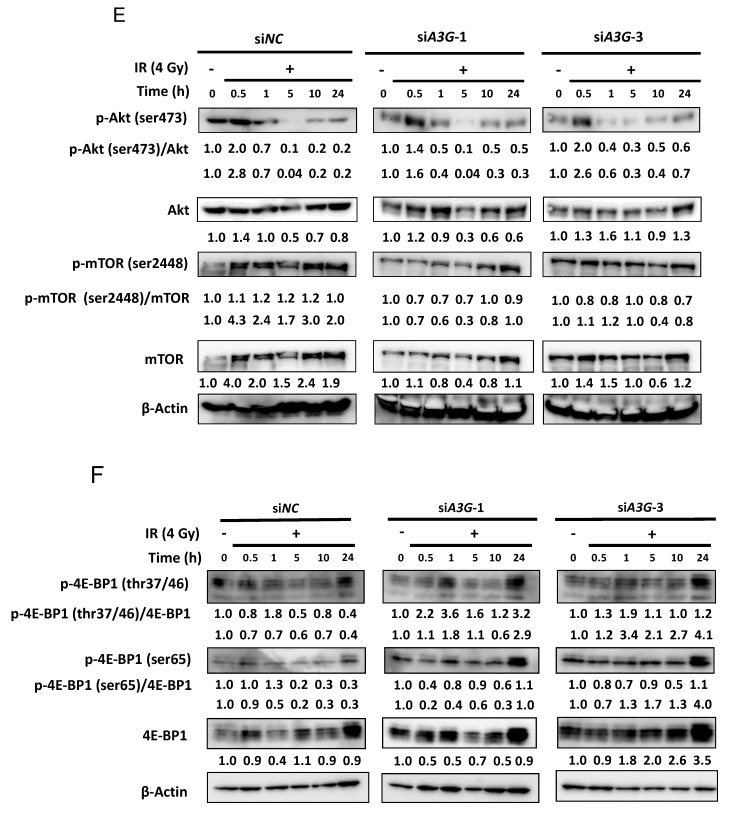
Radiosensitization effects of *A3G* knockdown on pancreatic cancer MIAPaCa2 cells and changes in cell cycle distribution and DNA damage response pathway. (**A**) The effect of *A3G* knockdown by a different siRNA (si*A3G-3*) on radiosensitization in pancreatic cancer MIAPaCa2 cell line after γ-irradiation by colony formation assay. (**B**) Effects of two different siRNAs (si*A3G-1* and si*A3G-3*) on cell cycle distribution 24 h after γ-irradiation. Asterisks show *p* < 0.05. (**C**–**F**) Effect of si*A3G-1* and si*A3G-3* on the expression of proteins related to DDR and cell proliferation using Western blot analysis. Relative expression levels of proteins first normalized by β-actin, then normalized again to no irradiation controls are shown under each panel. In (**C**), the values of normalization for *siA3G-1* are obtained by comparison with the second left-most lane in the left-most panel and shown with underlines. In addition, for phosphorylated proteins, the ratio of the phosphorylated protein/total protein was shown as indicated for (**D**–**F**).

We further analyzed the radiosensitization mechanism in pancreatic cancer MIAPaCa2 cells, which also showed a relatively higher ER_10_ value of 1.25 among the cell lines. Target sequences of two different siRNAs, *A3G-1* and *-3*, are located at exon 4 and exon 3–4, respectively. Four isoforms are registered as candidate splicing variants for the *A3G* gene: isoforms 1, 2, and 3, codes 384, 313, and 317 amino acids, respectively, while isoform 4 encodes a shorter peptide-lacking C-terminal catalytic domain (Figure S1). Two siRNAs, *A3G-1* and *-3,* knocked down the *A3G* mRNA level to less than 1% and 7%, respectively (Figure S2). We observed that knockdown of *A3G* with si*A3G-3* in MIAPaCa2 cells caused increased sensitivity to γ-irradiation by colony-formation assay (Figure 2A).

Twenty-four hours after γ-irradiation at 4 Gy, *A3G*-knockdown cells showed enhanced levels of cell cycle arrest at S-phase and decreased G2/M arrest (Figure 2B).

Western blot analysis after γ-irradiation at 4 Gy showed that a slight increase in PARP1 cleavage at 24 h post-irradiation suggested apoptosis had started to increase, although the level of cleaved PARP1 was not different among control (si*NC*) and knocked down cells at this stage. The γ-H2AX level remained higher 24 h after γ-irradiation in the two different siRNA knocked down (si*A3G-1* and si*A3G-3*) cells, suggesting that the DNA double-strand break (DSB) repair process was delayed (Figure 2C). Transient downregulation of phosphorylated H3 level was observed 5–10 h post-irradiation (Figure 2C), suggesting that the common peak of S-phase was approximately 5 h post-irradiation. γ-Irradiation induced phosphorylated CHK2 in all cases. Although the level decreased similarly 5–10 h post-irradiation in both the control and the knocked down cells, bands at 10 and 24 h were positioned at the slightly lower molecular weight in *A3G*-knockdown cells, suggesting the number of phosphorylated residues of CHK2 was lower (Figure 2C) at 24 h. However, the level of phosphorylated p53 at ser-15 was also higher after 0.5 h both in si*NC* and knocked down cells, and the ratio of the phosphorylated form to total p53 protein was also higher in all cases at 24 h post-irradiation (Figure 2D). Because the increase in γ-H2AX level, indicating delayed DSB repair, and the enhanced S-phase arrest were both observed, there is a possibility that ATR DNA damage response (DDR) pathway activation continued longer in the *A3G*-knockdown cells post-irradiation.

We also analyzed the effects on the PI3K cell proliferation signaling pathway (Figure 2E,F). The phosphorylated mTOR (ser2448) level did not show consistent changes among the cells after 4 Gy irradiation. On the other hand, the phosphorylated Akt level was augmented at 10–24 h in the knocked down cells (Figure 2E), and the downstream factor 4EBP1 phosphorylation both at Thr37/46 and ser65 showed a slightly higher level compared with control cells at 10–24 h post-irradiation (Figure 2F). These results suggest that *A3G* dysfunction causes an increased DDR defect involving enhanced DSB damage and cell cycle arrest at S-phase.

### 2.4. Effect of A3G Dysfunction in Lung Cancer A549 Cells

**Figure 3:**
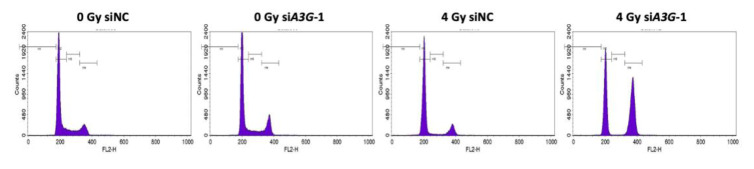

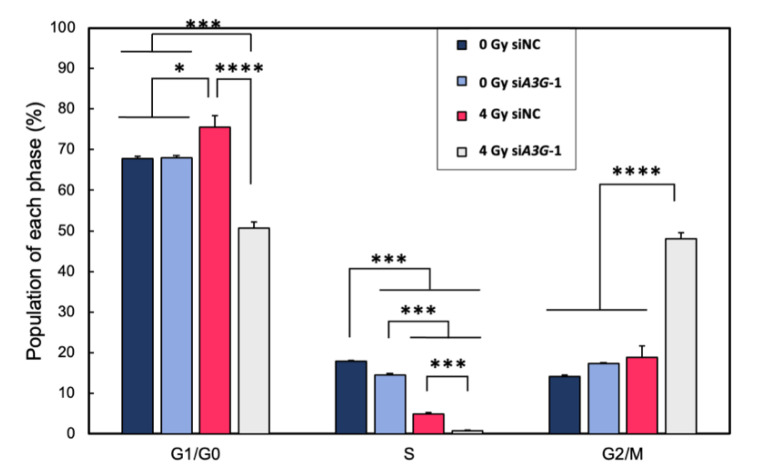
Radiosensitization effects of *A3G* knockdown on lung cancer A549 cells. (**Top**) Cell cycle analysis of *A3G-*knockdown A549 cells with *siA3G-1* on cell cycle distribution 24 h after γ-irradiation at 4 Gy. (**Bottom**) Population of cells in G0/G1, S, and G2/M phases. Values represent mean ± SE from 3 independent experiments. *, *p* < 0.05; ***, *p* < 0.001; ****, *p* < 0.0001.

As described above, the radiosensitizing effect induced by knockdown of *A3G* is suggested to be caused by DNA repair defects. We also compared the effects of *A3G* knockdown on cell cycle arrest after γ-irradiation in A549 cells, which showed the relatively lower *A3G* expression level (Figure 1C). As shown in Figure 3, twenty-four hours after irradiation, an approximately 2-fold increase in the G2/M phase percentage and a concomitant decrease in S-phase percentage were observed in *A3G*-knocked down cells compared with the control cells.

### 2.5. A3G Dysfunction Induces Radiosensitization in the Mouse Xenograft Model

**Figure 4:**
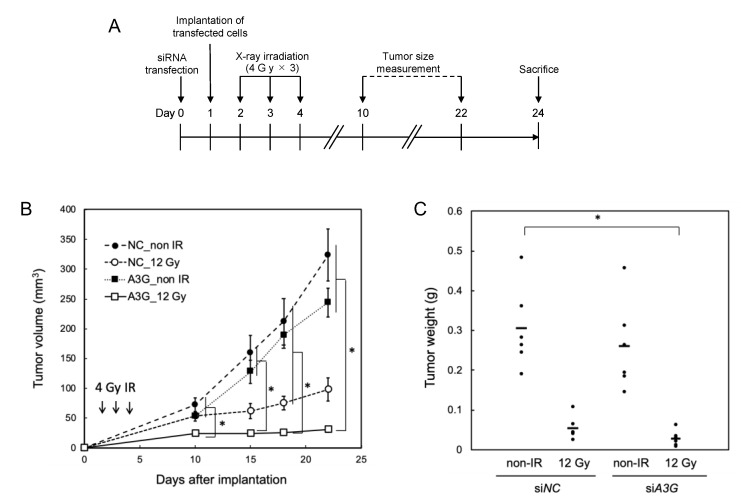
Radiosensitization effect of *A3G* knockdown on xenograft model of A549 cells. (**A**) Scheme of experiment. A549 cells were transfected with either the control (si*NC*) or si*A3G*-1 and were subcutaneously implanted with Matrigel in the left hind legs of nude mice and then local irradiation with X-ray at 4 Gy was carried out on days 1 to 3 after implantation. (**B**) Tumor volumes of non-irradiated mice and irradiated mice of control and si*A3G*-1 transfected groups. n = 6. * *p* < 0.05. (**C**) Tumor weights measured for each mouse in the groups. * *p* < 0.05.

A xenograft model was utilized to investigate whether *A3G* dysfunction suppresses the growth of X-ray-irradiated A549 tumors. A549 cells transfected with either the control or si*A3G*-1 were subcutaneously implanted in nude mice and then X-ray irradiated on days 1 to 3 after implantation (Figure 4). As shown in Figure 4B,C, the *A3G* knockdown itself slightly suppressed the tumor growth without the irradiation condition and further attenuated the tumor growth after three doses of 4 Gy X-ray irradiation, although a statistically significant difference was not observed between irradiated si*NC* control and irradiated *A3G*-knockdown groups. Tumor volumes in the irradiated *A3G*-knockdown group were uniformly small, while non-irradiated and irradiated control si*NC* and non-irradiated *A3G*-knockdown groups had variations in the tumor weight at 24 days after implantation. A statistical difference in tumor weight was only detected between non-irradiated si*NC* and irradiated *A3G*-knockdown groups (Figure 4C). This suggests that *A3G* knockdown suppressed tumor xenograft growth, possibly in a synergistic manner with X-ray irradiation.

## 3. Discussion

Radiotherapy is one of the most important approaches for cancer treatment and its importance is expected to increase because of its high therapeutic efficacy and low invasiveness. To increase the clinical benefits of radiotherapy, various methods have been improved and developed.

In this study, we observed 671 genes as radiosensitizing candidates from the genome-wide negative screening using A549 lung cancer cells. These candidate genes included known radiosensitizing targets, such as *PARP1*, *HSP90*, *HDAC*, and *ATM*, which demonstrated that the screening system worked as expected. In this study, we focused on *A3G*, a cytosine deaminase family protein, which is involved in DNA damage repair and protection from HIV infection [20].

In lymphoid cell lines, expressing a high *A3G* level is reported to be associated with efficient DSBs repair and enhanced cell survival after IR [23,24], suggesting that *A3G* expression levels can be a determinant of radiosensitivity in these cells. In contrast, our analysis with different types of solid cancer cell lines showed no correlation between *A3G* expression level and cell survival rate after γ-irradiation (data not shown). This indicates that *A3G* expression level would not be appropriate for a clinical marker for predicting response to radiotherapy for solid cancers. However, comprehensive correlation analysis across more diverse cancer cells could lead to finding the appropriate classification of cancer cells to utilize *A3G* expression level as a predictive marker for determining the benefit of radiotherapy.

The downregulation of *A3G* with siRNA enhanced radiosensitivity in 10 out of the 11 cancer cell lines tested, suggesting that targeting *A3G* can sensitize various cancer cells to radiation. Although *A3G* expression level is low in A549, HeLa, and SAS cells, they were sensitized to radiation by *A3G* knockdown. When MIAPaCa2 cells were knocked down with si*A3G*, the cells showed enhanced S-phase arrest and the disturbance of DNA DSB repair and p53-dependent responses. On the other hand, in A549 cells that show a relatively lower *A3G* level, increased G2/M phase arrest was observed. A3G is known to promote microhomology-mediated end joining (MMEJ)-directed DSB repair by inducing C to U mutations to ssDNA overhang generated during the repair process in lymphoma cells [23]. It was concluded that dysfunction of *A3G* induced the inhibition and delay of DNA repair after γ-irradiation, which caused the activation of cell cycle checkpoint, and resulted in either S-phase or G2/M arrest, depending on the cancer cell lines.

In a xenograft model of A549 cells, a combination of X-ray irradiation and *A3G* knockdown suppressed tumor growth, suggesting that A3G could be useful as a target for radiosensitization.

These results suggest that A3G may be a useful target not only in cancer cells with high A3G expression level but also in cells with low expression level. On the other hand, we found that osteocarcinoma-derived U2OS cells, which harbor a normal p53 pathway, did not show radiosensitization by *A3G* knockdown. The factors governing radiosensitization by the suppression of A3G remain to be elucidated. Specific inhibitors of A3G have not yet been developed. A3G inhibitors, which could be optimized for radiosensitization, may be useful for further investigation for clinical use.

## 4. Materials and Methods

### 4.1. Cell Culture

A549 (purchased from ATCC, Manassas, VA, USA) and PC14 (obtained from Dr. Hayata, Tokyo Medical College) cells were cultured in RPMI-1640 medium (Gibco Life Technologies Corp., Carlsbad, CA, USA). HeLa (obtained from National Cancer Center, Tokyo, Japan) and SBC-5 cells (obtained from Okayama University, Okayama, Japan) were cultured in Minimum Essential Medium (Gibco). A375 (purchased from ATCC), DU145 (purchased from RIKEN BRC, Tsukuba, Japan), MDA-MB-231 (purchased from ATCC), MIAPaCa2 (obtained from National Cancer Center), SAS (purchased from JCRB Cell Bank, Osaka, Japan), SW480 (purchased from JCRB Cell Bank), and U2OS (purchased from ATCC) cells were cultured in Dulbecco’s Modified Eagle’s Medium (Gibco). All media contained 0.2% NaHCO_3_ (Wako, Osaka, Japan), 10% FBS (Hyclone, Cytiva, Tokyo, Japan) and 1% penicillin–streptomycin (Invitrogen, Waltham, MA, USA). All cells were cultured at 37 °C in a humidified atmosphere maintained at 5% CO_2_.

### 4.2. γ-Irradiation and X-ray Irradiation

γ-Irradiation was performed with ^137^Cs γ-irradiator at the National Cancer Center Research Institute (at 100 cGy/min, Gammacell 40 Exactor, Best Theratronics, Ottawa, Ontario, Canada) and at Nagasaki University (PS-3100SE, Pony Industry, Osaka, Japan). For X-ray irradiation, X-ray Biological Irradiator (0.33 Gy/min; CP-160, Faxitron X-ray Corp., Wheeling, IL, USA) was used at the National Cancer Center Research Institute.

### 4.3. Negative Screening Using an shRNA Library

The negative screening was performed using the Decode RNAi Annotated Genome Screening Library: Negative Selection Kit (Thermo Scientific, Waltham, MA, USA) according to the manufacturer’s protocol. A549 cells were infected with a lentiviral siRNA expression library using TransDux (System Biosciences, Palo Alto, CA, USA) (pool 1 and pool2). Two days after infection, noninfected cells were removed by three-day treatment with puromycin, and cells were divided into two groups, one of which was irradiated with 4 Gy γ-ray, the other was stored as non-irradiated control. Seven days after irradiation, genomic DNA was purified using DNeasy Blood & Tissue Kit (Qiagen, Hilden, Germany). Barcode sequences incorporated into genomic DNA were amplified by PCR using primers included in the kit and labeled with cyanine-3 for non-irradiated control, with cyanine-5 for the irradiated group. After purification with Amicon Ultra-0.5 Centrifugal Filter Devices (Millipore, Burlington, MA, USA), the labeled sequences were hybridized with a microarray slide for 17 h at 65 °C in ICES/NMB-001 (Agilent Technologies, Santa Clara, CA, USA). Signals from cyanine-3 or cyanine-5 were measured with DNA Microarray Scanner/Agilent Scan Control Software (Agilent Technologies).

### 4.4. siRNA Transfection

Prior to transfection, 2 × 10^5^ cells were seeded into 6-well plates. Cells were transfected with a DsiRNA targeting *A3G* (Integrated DNA Technologies, Coralville, IA, USA) using Lipofectamine RNAi MAX reagent (Life Technologies, Carlsbad, CA, USA), according to the manufacturer’s protocol. The DsiRNAs for A3G were purchased from Integrated DNA Technologies (si*A3G-1*, si*A3G-3*, Table A1). DsiRNA was used at a final concentration of 20 nM or 10 nM in Opti-MEM. Negative Control DsiRNA (si*NC*, Integrated DNA Technologies) was used as a negative control.

### 4.5. Gene Expression Analysis (Quantitative RT-PCR)

RNA was prepared from each cell line with High Pure RNA Isolation Kit (Roche, Basel, Switzerland). Extracted RNA was reverse transcribed using High Capacity cDNA Reverse Transcription Kit (Applied Biosystems, Waltham, MA, USA) with RNase inhibitor (Applied Biosystems). The qRT-PCR analysis was performed using SYBR Select Master Mix (Life Technologies) with the CFX96 Real-Time System (Bio-Rad, Hercules, CA, USA). The mRNA levels were normalized to *GUSB* mRNA. The sequences of primer pairs are listed in Table A2.

### 4.6. Clonogenic Survival Assay

In 6-well plates, 2 × 10^5^ cells were seeded and DsiRNAs were transfected. These cells were reseeded in triplicate onto 6-well plates 24 h after transfection and irradiated 15 h after reseeding. Ten days after γ-irradiation, the colonies were fixed with 4% neutralized formalin (Wako) and stained with 0.01% crystal violet. Colonies consisting of more than about 50 cells were counted.

### 4.7. Cell Cycle Analysis

Cells were fixed with 70% ethanol, stained with dye solution containing 50 μg/mL propidium iodide (PI) (Sigma-Aldrich, Burlington, MA, USA) and 0.1 mg/mLRNase A (Sigma-Aldrich), and then analyzed using FACSCalibur (Beckton and Dickinson, Mountain View, CA, USA).

### 4.8. Western Blot Analysis

Western blotting was carried out as described elsewhere. Cells were harvested after washing with cold PBS(-) and lysed in Laemmli’s buffer. Proteins were transferred onto a polyvinylidene difluoride membrane and probed with the appropriate primary antibodies. The membrane was then incubated with corresponding horseradish peroxidase-conjugated secondary antibodies and the antigen–antibody complexes were visualized using Immobilon Western Chemiluminescent HRP Substrate (Millipore). Details of the antibodies used are provided in Table A3.

### 4.9. Animal Experiments

A549 cells were transfected with the A3G or negative control DsiRNA 1 day before implantation. The following day, 1 × 10^6^ transfected cells were suspended in Matrigel (Corning, Corning, NY, USA) and subcutaneously injected into both hind legs of 6-week-old male BALB/c nude mice (Japan SLC, Inc., Tokyo, Japan). Six mice were injected with siRNA-treated cells. On days 1 to 3 after implantation, the mice were subjected to daily X-ray irradiation (4 Gy). By protecting the entire body except the left leg with a lead shield (5 mm thickness), irradiation was limited to the left hind leg only. Tumor volumes were measured with micrometer calipers and calculated as follows: volume = pi/6 × (smallest diameter)^2^ × (largest diameter). Twenty-four days after implantation, mice were sacrificed and tumor weights were measured. All animal studies were approved by the Animal Experimental Committee of the National Cancer Center (Code no. T14-004) and were performed in accordance with the Guidelines for Animal Experiments of the National Cancer Center, which meet the ethical guidelines for experimental animals in Japan.

### 4.10. Statistical Analysis

Statistical analysis was carried out by Student’s *t*-tests using JMP (SAS Institute Inc., Cary, NC, USA).

## Figures and Tables

**Table 1:** The enhancement ratios at 10% survival (ER_10_) to cell lines.

Cell Line	ER_10_	Disease	Species
A549	1.35	Lung adenocarcinama	Human
DU145	1.28	Prostate carcinoma	Human
MIAPaCa2	1.25	Pancreatic ductal adenocarcinoma	Human
SW480	1.23	Colon adenocarcinoma	Human
MDA-MB-231	1.2	Breast adenocarcinoma	Human
SAS	1.17	Tongue squamous cell carcinoma	Human
SBC5	1.16	Lung small cell carcinoma	Human
A375	1.14	Amelanotic melanoma	Human
PC14	1.12	Lung adenocarcinoma	Human
HeLa	1.1	Human papillomavirus-related endocervical adenocarcinoma	Human
U2OS	1.01	Osteosarcoma	Human

## Data Availability

The data that supported the findings of the results are available from the corresponding author upon reasonable request.

## Supplementary Materials

The following supporting information can be downloaded at: https://www.mdpi.com/article/10.3390/ijms23095069/s1.

## Appendix A

**Table A1:** Sequence of siRNAs.

Gene	Forward (5′→3′)	Reverse (5′→3′)
siRNA1 for *A3G*	GGAAUAAUCUGCCUAAAUAUUAUAT	AUAUAAUAUUUAGGCAGAUUAUUCCAA
siRNA3 for *A3G*	AGAUCAUGAAUUAUGACGAAUUUCA	CUUCUAGUACUUAAUACUGCUUAAAGU

**Table A2:** Primers for qRT-PCR.

Gene	Forward (5′→3′)	Reverse (5′→3′)
Primers set 1 for *A3G*	CCGTCTGGCTGTGCTACGAA	ACGATGCAGCTTCCTCCACT
Primers set 2 for *A3G*	CCCTGACCATCTTTGTTGCC	CGAACTTGCTCCAACAGTGCT

**Table A3:** Antibodies used in this study.

Antibody	Company	Catalog#	Species	Dilution	Purpose
PARP	CST	9542	H,M,R,Mk	1/1000	WB
p-P53 (ser15)	CST	9284	H,M,R,Mk	1/500	WB
P53	CST	9282	H,Mk	1/1000	WB
p-Chk2 (thr68)	CST	2197	H	1/1000	WB
γ-H2AX (ser139)	CST	9718	H,M,R,Mk	1/1000	WB
p-HistoneH3 (ser10)	GeneTex	128116	H	1/1000	WB
p-mTOR (ser2448)	CST	2971	H,M,R,Mk	1/1000	WB
p-Akt (ser473)	CST	9271	H,M,R,Mk,Dm	1/1000	WB
Akt	CST	4691	H,M,R,Mk,Dm	1/1000	WB
p-4E-BP1 (thr37/46)	CST	2855	H,M,R,Mk,Dm	1/1000	WB
p-4E-BP1 (ser65)	CST	9451	H,M,R,Mk	1/1000	WB
4E-BP1	CST	9644	H,M,R,Mk	1/1000	WB
β-Actin	Sigma-Aldrich	A2228	H	1/10,000	WB
