# Supplementary figures and images for: Radiosensitization to γ-Ray by Functional Inhibition of APOBEC3G
Source: Int J Mol Sci. 2022 May 3;23(9):5069. doi: 10.3390/ijms23095069 (PMC9100529; doi:10.3390/ijms23095069)

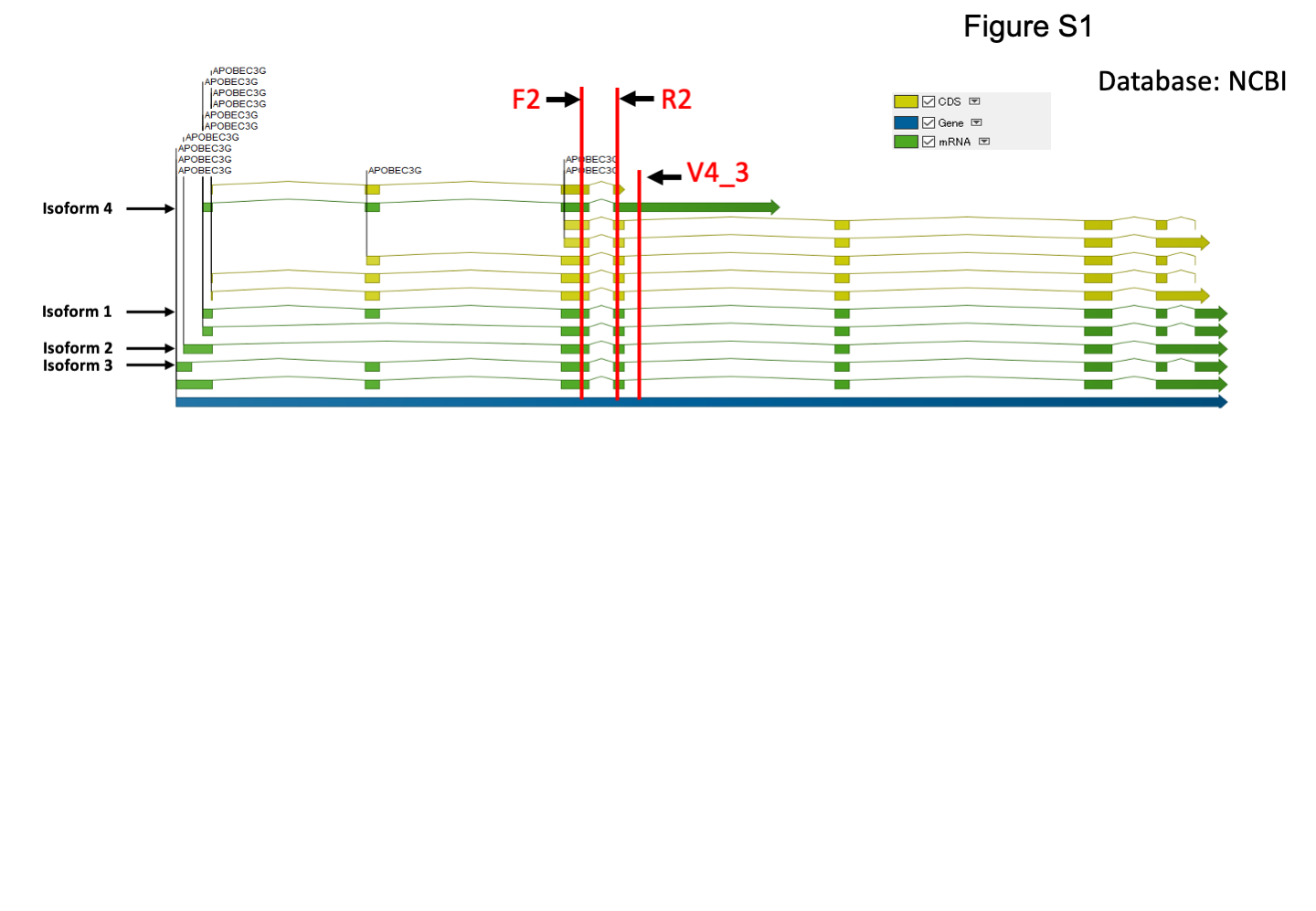

Supplement: Supplementary file 1 [file ijms-23-05069-s001.zip › Figure S1.tiff]

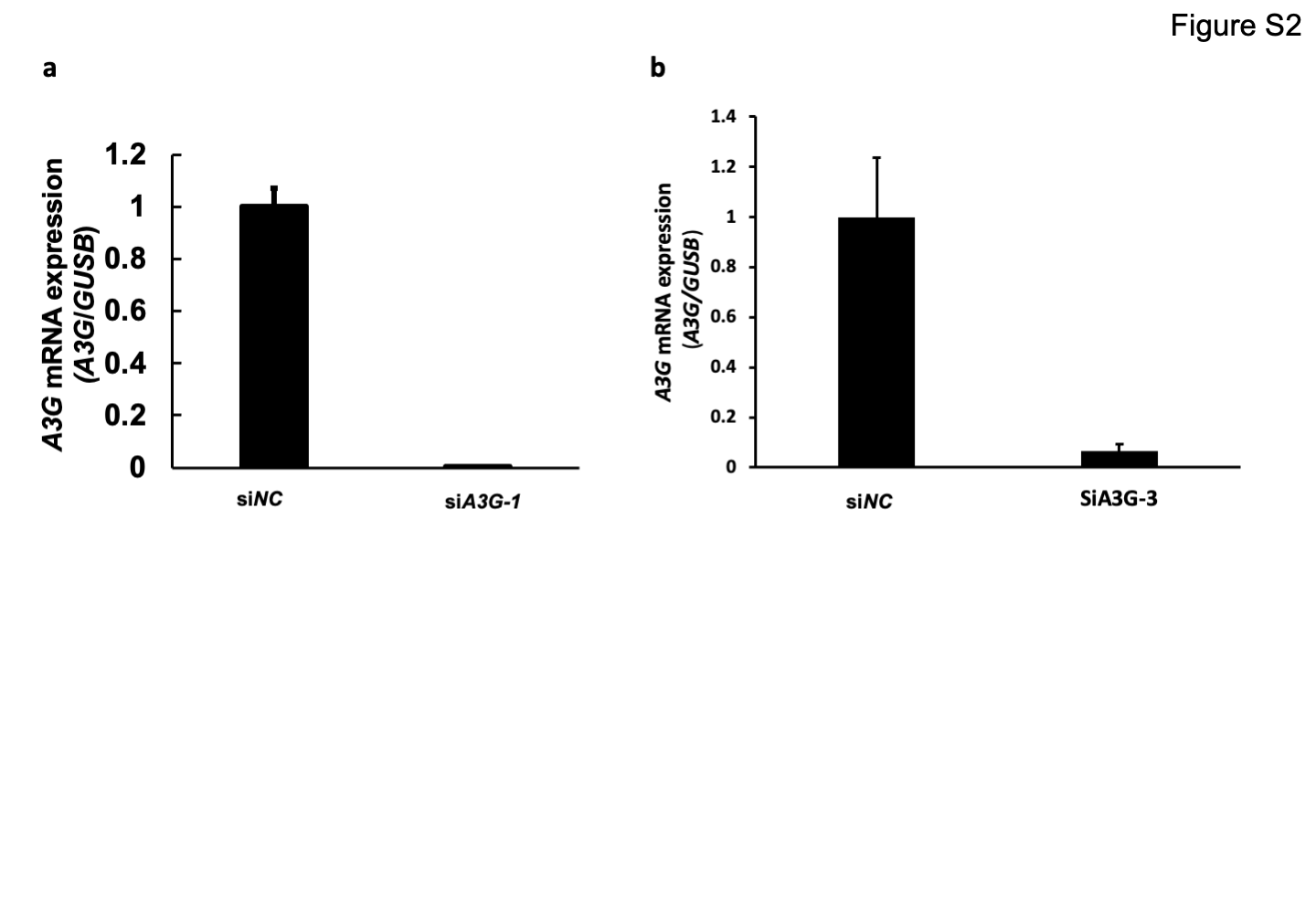

Supplement: Supplementary file 1 [file ijms-23-05069-s001.zip › Figure S2.tiff]
